# Bread as the Staple Food

**Published:** 1917-04-21

**Authors:** 


### FOOD IN WARTIME.*

### Bread as the Staple Food.

It is largely owing to laziness that bread has come to assume so preponderating a place in the institution dietary. Bread is the easiest of all foods with which to satisfy hunger and provide for the body's requirements. It needs no cooking. For the last two generations it has been very cheap. It needs but the slight aid of a little fat or sweet relish to make it palatable. It keeps well. And to sum up all, it gives the minimum of trouble to everyone concerned. And thus for the poor mother of a family who is totally ignorant of food preparation or food values, as well as for the managers of vast institutions with every culinary resource at their disposal, bread has come to be considered the staple article of diet. Now there comes a scarcity of bread, and it is no bad thing for all in this country that they are compelled to open their eyes and reflect whether after all it is rational or desirable that this monotonous system of dieting should continue.

The Birmingham Guardians have laid down the principle that 6 lb. of bread, including flour, per week is essential for children. They do not appear to realise not only that this amount is actually not available at the present time, but that this has never been a satisfactory way of feeding children. Dietaries which depend on bread and butter or margarine right through the day, with chunks of bread also at dinner, must be thoroughly overhauled. The cook must do more, the housekeeper must use her brains. And the household will rejoice, for, after all, is not the incessant bread-and-butter diet to which many workers in institutions are treated responsible for much indigestion and depression? Every meal should be a time of refreshing, a little feast with some slight element of surprise in it.

In many hospitals where the dietary is varied the average amount of bread consumed by the household and patients taken altogether does not exceed the war ration of half a pound a day. At the Radcliffe Infirmary, Oxford, the average weight in pounds consumed per head per day worked out as follows :

| 1916 | 1915 | 1914 | 1913 |
|---|---|---|---|
| 0.47 | 0.48 | 0.49 | 0.46 |

At the Norfolk and Norwich Hospital the amount used in 1916 was 210 lb. per head, or an average of about 9 oz. a day. We have now the welcome pronouncement of Lord Devonport, *re* hotel rations, that these large establishments may requisition for total numbers without worrying about whether individuals get less or more than their allowance.

It cannot be doubted that institutions are entitled to use the same liberty, and to receive sufficient supplies for the week to cover an average of $3\frac{1}{2}$ lb. of bread and $\frac{1}{2}$ lb. of flour for every person boarded. It is, therefore, clear that, by the exercise of a small amount of ingenuity, the bread ration will be ample for the needs of the hungriest member of the staff. But in order to remove scruples it would be wise to give everyone the choice of adhering somewhat rigidly to the scale. If something even be saved on the bread ration, all the better. One might go farther and assert that everyone in the present emergency should study to bring their ration down *below* the maximum.

Institution loaves are nearly always baked to contain 4 lb. in weight, and are shaped in rectangles for the convenience of cutting by machine. It will be found that a 4-lb. loaf will usually cut into forty-two squares, including crusts at either end, and these squares are served cut in half. Four of these half-slices will weigh 3 oz., so that ten half-slices are well within the ration of $\frac{1}{2}$ lb. a day. It would not be a bad plan to test this calculculation on the loaves as supplied and let the result be written on a card to be placed on the table for all to see. There are not many persons who want to eat more than ten slices of bread a day, unless they really get very little else on which to satisfy their appetites. But if bread be the only breakfast dish, if bread be served at 10.30 for lunch, if bread be distributed round the table at dinner, if bread and butter appear again for tea, and supper consists mainly once more of bread and cheese, then it is hard to see how the ration can last out.

The remedy lies in providing a substitute. Oatmeal porridge should be the staple breakfast dish, and will make its way to popularity if properly prepared. Three or four slices of bread in addition will prove enough for most appetites. At the intermediate lunch oat-cake or oatmeal biscuits would be an attractive novelty. At dinner the conventional slice of bread ought to disappear altogether while this crisis lasts. It is purely a matter of habit to nibble bread through this meal, and if the courses are as satisfying as they ought to be for persons engaged in active work there should be no need to supplement with bread, which is tantamount to a confession of failure in the menu. At afternoon tea barley-scones might take the place of bread and butter. We append a recipe for these easily-made delicacies. With a good thick soup for supper three or four more slices of bread may be enjoyed.

* Previous articles appeared on February 24, March 10, 24, and 31, and April 14.

And at the end of the day there will thus be some of the ration spared for the nation.

*Barley-meal Scones.*—Add a little sugar and mix the barley-meal with hot milk to a thick paste. Roll out thin and cut into the required shapes. The scones should then be baked in a quick oven or on a griddle over a hot fire or gas-stove. When eaten hot they should be split and a small nut of margarine inserted in each. They are also very good cold. A little salt may be substituted for sugar.

*Oat Cake.*—Few articles of diet are cheaper or more easily prepared than this. The principal requirement is a pan or slab of iron or stone, in shape somewhat like a very solid frying-pan. Two or three handfuls of oatmeal are well mixed with cold water and a seasoning of salt. The mixture is then kneaded well with the hands for some time till it is of the right consistency. It is then rolled out on a board in the required shape, as thin as possible, care being taken to strew it on both sides with fine meal to obviate stickiness. It is transferred at once to the pan and cooked over a bright fire for a few minutes, first on one side and then on the other. It is better, if a large batch is to be prepared, to mix only so much at a time as can be baked at once. The cakes keep well, but are nicer if freshened up over the fire shortly before use.

*Rice Flour* is an excellent substitute for wheaten flour in making both cakes and bread. In pre-war days rice flour was a common and harmless adulteration in bread, and its use would materially assist in keeping down the amount of flour required in institutions. It can be used for making pastry and for bread sauce, and, in fact, there is hardly any department of the culinary art in which rice flour may not be pressed into the service. Its use is greatly facilitated by mixing it with wheaten flour, which will thus go twice as far. It cannot be too well understood that one main problem in serving war rations is to satisfy the appetite, and managers are making a great mistake when they bring down the quantities served below what is required for this purpose.

